# Collaborative care for mental health: a qualitative study of the experiences of patients and health professionals

**DOI:** 10.1186/s12913-020-05691-8

**Published:** 2020-09-09

**Authors:** Jorun Rugkåsa, Ole Gunnar Tveit, Julie Berteig, Ajmal Hussain, Torleif Ruud

**Affiliations:** 1 Health Services Research Unit, Akershus University Hospital, 1478 Lørenskog, Norway; 2 Centre for Care Research, University of South-Eastern Norway, Porsgrunn, Norway; 3 R&D Department of Mental Health, Akershus University Hospital, Lørenskog, Norway; 4 Institute of Clinical Medicine, University of Oslo, Oslo, Norway; 5 Department of Acute Psychiatry Oslo University Hospital, Oslo, Norway; 6 Division of Mental Health Services, Akershus University Hospital, Lørenskog, Norway

**Keywords:** Shared care, Integrative care, Collaborative care, Mental health care, GP mental health care, Community mental health care, Community Mental Health Centre

## Abstract

**Background:**

Health policy in many countries directs treatment to the lowest effective care level and encourages collaboration between primary and specialist mental health care. A number of models for collaborative care have been developed, and patient benefits are being reported. Less is known about what enables and prevents implementation and sustainability of such models regarding the actions and attitudes of stakeholders on the ground. This article reports from a qualitative sub-study of a cluster-RCT testing a model for collaborative care in Oslo, Norway. The model involved the placement of psychologists and psychiatrists from a community mental health centre in each intervention GP practice. GPs could seek their input or advice when needed and refer patients to them for assessment (including assessment of the need for external services) or treatment.

**Methods:**

We conducted in-depth qualitative interviews with GPs (*n* = 7), CMHC specialists (*n* = 6) and patients (*n* = 11) in the intervention arm. Sample specific topic guides were used to investigate the experience of enablers and barriers to the collaborative care model. Data were subject to stepwise deductive-inductive thematic analysis.

**Results:**

Participants reported positive experiences of how the model improved accessibility. First, *co-location* made GPs and CMHC specialists accessible to each other and facilitated detailed, patient-centred case collaboration and learning through complementary skills. The threshold for patients’ access to specialist care was lowered, treatment could commence early, and throughput increased. Treatment episodes were brief (usually 5–10 sessions) and this was too brief according to some patients. Second, *having experienced mental health specialists in the team* and on the front line enabled early assessment of symptoms and of the type of treatment and service that patients required and were entitled to, and who could be treated at the GP practice. This improved both care pathways and referral practices. Barriers revolved around the organisation of care. Logistical issues could be tricky but were worked out. The biggest obstacle was the funding of health care at a structural level, which led to economic losses for both the GP practices and the CMHC, making the model unsustainable.

**Conclusions:**

Participants identified a range of benefits of collaborative care for both patients and services. However, the funding system in effect penalises collaborative work. It is difficult to see how policy aiming for successful, sustainable collaboration can be achieved without governments changing funding structures.

**Trial registration:**

ClinicalTrials.gov identifier: NCT03624829.

## Background

General Practitioners (GPs) are the sole clinicians for up to 95% of patients with mild to moderate mental health disorders, such as depression and anxiety [1, 2]. This is in line with health policy in many countries, including Norway, which directs treatment activities to the lowest effective level [3]. However, there are several reasons why this might be problematic when it comes to mental disorders. Studies report that GPs detect between 25% [4] and 50% of cases [5] of mental disorders among patients who has been diagnosed independently as meeting diagnostic criteria. Treatment commonly used in specialist care, such as psychotherapy, multidisciplinary care and regular calibration of psychotropic medication, is usually outside the scope of core GP training [6, 7]. Treatment of mental disorders is also usually time consuming. GPs often struggle to balance care for this patient group against their other responsibilities [8, 9], and there are few monetary incentives for them to focus on mental health [6]. GPs often experience that they have limited access to consultations with mental health specialists [6, 10–14]. Those with enhanced access to such collaboration, however, report improved skills, knowledge and confidence to treat mental disorders [15–17].

To address these issues and offer better care, various models for how primary and specialist mental health care can work together have been developed and evaluated in different countries [18–21]. The scope of such models varies from simple methods to improve communication between care levels to fully integrated services [22]. Following a recent position paper, in this article we apply the broad term *collaborative care* to describe “the process whereby primary care and mental health providers share resources, expertise, knowledge and decision-making to ensure that primary care populations receive person-centred, effective and cost-effective care from the right provider in the most convenient location and in the most timely and well-coordinated manner” [21]. Important components in collaborative care include the establishment of cooperative relationships with clarified professional roles and enhanced interprofessional communication, structured organization and follow-up, and co-location (i.e., collaborative team members work at the same physical location) [18, 22, 23]. Most models also include care coordination [24, 25]. A 2012 Cochrane review found that while study results vary, in general, patients treated for depression and anxiety with collaborative care improve more than controls in the short, medium and long term. There is also evidence of benefit in terms of appropriate use and adherence to medication, mental health-related quality of life, and patient satisfaction [20]. However, as models vary considerably regarding which components they include [26], it can be difficult to ascertain what the ‘active ingredients’ really are [18, 20, 24, 27].

To understand the outcome of complex interventions, that is interventions with several interacting components, it is important to consider the attitudes and actions of stakeholders “on the ground” as they can shed light on whether and how interventions work [28]. We found two systematic reviews of qualitative studies that identify enablers and barriers to collaborative care, one focused on depression [25] and the other on anxiety and depression [26]. Stakeholders’ attitudes to collaboration, lack of understanding of each other’s roles and responsibilities, and training issues were identified as key barriers along with more practical issues such as lack of space for extra staff, compatibility of IT-system and issues of reimbursement. Enablers included having local GP champions and regular feedback on progress. Furthermore, co-location facilitated face-to-face interactions that improved interprofessional communication and focused it on concrete cases. The social skills (e.g., having relationship building skills, being engaged and visible) and professional skills (e.g., expertise and clinical experience) of mental health professionals were crucial for embedding models into the primary care setting. These findings notwithstanding, both reviews conclude that while the evidence base for collaborative care seems strong, further work is needed in order to understand how models can be mainstreamed. Specifically, qualitative studies that include patients, front-line staff and managers [25], and that represent settings beyond the US and UK [26] are needed.

Few studies have specifically investigated patients’ experiences of collaborative care. One recent review identified that although experiences vary – possibly due to model variability – patients value the opportunities collaboration holds for improving access, collaborative partnerships and patient-centred care [29]. The review concluded that further evaluative research should include patients’ perspectives.

To address current gaps in the literature, we report from a qualitative sub-study of a large research programme that adapted and implemented the Hamilton Family Health Team model for collaborative care (the HFHT-model) in a Norwegian setting [30]. The original model, developed and used in Canada for over 20 years, has been described in detail previously [31, 32]. In brief, the HFHT-model involves placing various health professionals within existing GP practices to form Family Health Teams that can then offer coordinated mental and somatic health care. These health professionals include nurses or other health professionals with training in psychiatric care, physiotherapy, nutrition, pharmacology or occupational therapy. Their involvement should enhance GPs’ ability to treat more patients, reduce service fragmentation and improve the treatment of co-morbidities. Co-location in primary care is intended to promote collaboration and provide care close to where patients live. Evaluations have shown positive intended effects [33] and high levels of satisfaction [15]. This collaborative care model is well aligned with the current Coordination Reform in Norway, which has as an explicit aim to improve collaboration between service levels [3]. We tested the effectiveness of this approach in a cluster randomised control trial (RCT) in which three GP practices were randomised to adapt and implement the HFHT-model in their local context together with the relevant specialist service, and with three practices serving as controls. Details of the intervention and the cluster-RCT are available [30, 34]. The qualitative study reported here was designed to identify, from the perspectives of patients and health professionals, the collaborative care model’s advantages and disadvantages as well as its enablers and barriers.

## Methods

### Study setting

The intervention was situated in three boroughs in the eastern part of Oslo, Norway. In Norway, primary mental health care is the responsibility of local authorities (municipalities and boroughs) and specialist care is provided through state-owned health trusts. In addition to acute hospital wards, the trusts provide services through specialist community mental health centres (CMHCs) with outpatient clinics, inpatient wards and some mobile outreach services. While local authorities provide support through multidisciplinary mental health teams, self-help groups, family support and (in some places) psychological services, most patients receive mental health care from their GP. Norway operates with a list-patient system in general practice, by which all inhabitants are attached to a GP of their own choosing. The system is funded by a combination of a capitation component based on the number of patients on the GP’s list (the average being just over 1100), a fee-for-service component (reimbursed by the state), and relatively modest co-payments by patients. Most referrals to specialist mental health services come from GPs, and GPs can also refer to services offered by the local authority.

### The intervention

Following a study visit to Hamilton, Ontario, in September 2015, the HFHT-model was adapted by the local services themselves and implemented in the period March 2016–November 2017. The teams were to be co-located at the GP practice, and it was decided that the CMHC clinicians involved should be experienced specialists, at consultant level, so that their level of expertise matched, and was complementary to, that of the GPs. The CMHC allocated 50% of the time of three Consultant Psychologists (one in each intervention practice) and one Consultant Psychiatrist throughout the project period. The GP practices provided office space and support staff. The CMHC specialists were given user accounts and access to the GPs’ IT and records systems. During the project period, the GPs sought input or advice from the CMHC specialists when needed and referred patients to them for assessment (including assessment of need for external services) or treatment. The GPs could book patient appointments directly in the psychologists’ calendar and the psychiatrist visited the practices at set times each week and was available by phone outside those times.

These factors were the backbone of the intervention, and beyond this, the three teams adapted their work to suit the local circumstances. For instance, one team established weekly meetings with partners in the borough’s services, while in the other two, external services were brought in as needed. The bulk of the cases where the GPs sought the involvement of CMHC specialists related to patients with moderate mental health problems such as anxiety (including PTSD) and depression. Some were also new patients with more severe problems such as borderline personality disorder, negative effects of complex trauma, and psychotic disorders.

### Design, sampling, and data collection

We conducted semi-structured individual interviews with three stakeholder groups at the end of the intervention period, when experiences were still fresh. The researchers invited the involved CMHC specialists, including the relevant managers (*n* = 7) and all GPs in the intervention practices (*n* = 10), to take part. Patients were sampled from those who had met with CMHC specialists at their GP practice. We asked the psychologists to identify patients with different characteristics in terms of age, gender, diagnosis, socioeconomic background, and perceived levels of satisfaction with the service, in order to capture a wide range of experiences [35]. After obtaining initial agreement, the psychologist forwarded contact details to JB who contacted the patient by phone to set up the interview.

We developed topic guides for each sub-sample, based on the literature and experiences from the intervention. The topics discussed with health professionals and patients were similar, with focus on delivering and receiving services, respectively, during the intervention. Participants were encouraged to speak freely, and the interviewer probed throughout for examples or asked for clarification and elaboration.

Interviews took place between December 2017 and March 2018. JR (senior researcher, female) interviewed 6 CMHC specialists, 3 men and 3 women. These were the 4 CMHC specialists who worked in the intervention teams and 2 managers. OGT (PhD student, male) interviewed 7 GPs, 4 men and 3 women with between 6 and 32 years of GP experience. Of 25 referred patients, 14 did not respond when contacted or declined. JB (trainee psychiatrist, female) interviewed 11 patients, 7 men and 4 women. They varied in age from 22 to 63 and suffered from anxiety, depression (and the combination of the two), ADHD, personality disorders, or acute stress as a result of life crises. The clinical participants were interviewed at their place of work, while patients were interviewed (by their own choice) in their homes.

The interviews lasted between 20 and 80 min. The role of the interviewer (none of whom were involved in model implementation or in treatment of the patients) was explained at the beginning of each interview, and participants were assured that identifiable information about them would not be disclosed. Only the interviewee and the interviewer were present during the interviews, which were recorded digitally and transcribed verbatim. The relevant interviewer then checked each transcription against the recording for accuracy and changed all names of individuals and organisations to agreed codes to make them anonymous.

### Analysis

All data were subject to thematic analysis [36] in inductive-deductive cycles. As we specifically wanted to investigate enablers and barriers to the adapted HFHT-model and participants’ experiences of how it worked, the first step in the analysis took a largely deductive approach. Based on the research questions and a read-through of each transcript, we identified broad codes to be applied to all three data sets. Examples of these codes include ‘views of what worked’, ‘internal collaboration’, ‘external collaboration’, ‘referral practice’ and ‘learning’. Not all codes applied to the patient sample. Each data set was coded separately (by the researcher who had carried out the interviews), using the agreed coding framework. In the second analytic step, we took a largely inductive approach to analysing the coding reports from step one. Data were broken down further for each sample separately to identify specific sub-themes. In the third step, we juxtaposed the resulting sub-themes. An overarching theme of ‘improved accessibility’ with a range of sub-themes emerged for the success and enablers, while ‘organisation of care’ became the overarching theme for the barriers identified. We use the themes and sub-themes as headlines in our presentation of results, and we include quotes from participants to illustrate and validate interpretations [37]. To protect anonymity, quotes are identified with sub-sample and interview number only. Unless specifically stated, quotes are selected to represent general themes and opinions in the relevant sub-sample.

## Results

### Model enablers and success: improved accessibility

When discussing the enablers and success of the adapted HFHT-model, the issues raised by patients, GPs and CMHC specialists surrounded *improved accessibility*. This was seen as resulting from two main model components: *co-location* and *having experienced mental health specialists in the team*, each with related sub-themes, as we describe next.

### Co-location

#### GPs and CMHC specialists being accessible to each other facilitate detailed, patient-centred case collaboration

Being present two full working days each week helped to embed the psychologists into the working life of the clinics. Some GPs pointed out how the social and professional skills of CMHC specialists enabled them to fit in and forge new working relationships. Working in the same environment facilitated close ongoing collaboration based on individual patient needs. In practice, this often took place as unplanned, opportunistic meetings as and when issues arose, such as “5 minutes in the corridor” or over lunch:

*There has been very close cooperation around these patients, and I have to say that’s the most striking thing about this project … Yeah, and there has been a lot of, like, informal discussion with the GPs after appointments, like, 5 min here and there, just to clarify things, divide up tasks … We maybe haven’t had the long case discussions that we have at the CMHC.* (CMHC 4).

Most of the patients experienced that the close communication helped their GP to be more involved in their mental health care:

*I feel my GP has been – maybe to a greater extent – involved in that. Not like, not because she’s been present or anything, but because I know that [the psychologist] and definitely that the psychiatrist and my GP have been in regular contact. And I went to her pretty – or much more – regularly the year I saw [the psychologist] so I experience that as positive.* (Patient 9).

While close collaboration was seen as positive by most patients, one said that it “felt strange” to talk to the psychologist with his GP present, possibly out of fear it would impact their existing relationship.

Most patients emphasised that it was important to them that all those involved in their care had access to their records. Sharing the electronic records system gave the psychologists instant access to historic and contextual information about the patients. They could add detailed notes about treatment activities and medication regimes, which then helped GPs to maintain them. Many of the GPs, who were used to lengthy referral processes to access specialist input, found that having “instant access” to the CMHC specialists was invaluable. In some cases, it made GPs refrain from prescribing antidepressants:

*There was maybe less talk about medication, which I think is good. You know, that you can, because if, the GP is often very much on his own [in decision-making] so in a way I think it’s … it’s medication that you end up with maybe, during a busy working day. (GP2).*

Several of the clinicians expressed the view that the complementary skills in the team made them better able to deal with co-morbidities. For instance, if a patient suffered from both panic attacks and heart disease, they could jointly arrive at an appropriate level of exercise that could alleviate psychiatric symptoms while not endangering cardiovascular health. There were also cases where it was agreed that depression treatment should be paused for a period while a patient was assessed for somatic illness.

#### Lowering the threshold makes mental health services more accessible to patients

The patients particularly valued that they could access mental health services without having to “fight” for them. As GPs could book consultations directly into the psychologists’ diary during patient consultations, treatment by a specialist could commence within days. Patients, GPs and CMHC specialists all agreed that patients benefitted from getting help for their mental health problems close to where they lived and at a place they were familiar with. Some patients expressed relief that they could get help without attending the CMHC where they risked being recognised as someone with a mental health problem. This fear of stigma was echoed in some GPs and CMHC specialists' impressions of patients’ concerns. Other patients stated, however, that location was secondary to the personality of the clinician and the only patient who had previously been treated at the CMHC said he had not experienced this as stigmatising.

The very different ramifications surrounding the usual practice of both GPs and CMHC specialists was acknowledged, including the nature of the time pressures they were under. Moving some of the flexibility from the specialist service into the GP practice was recognised as another way in which the model lowered the threshold and facilitated early access and intervention:

*There’s really a difference between the boundaries that a psychologist and a GP work within […*] *You can’t expect a GP to achieve in 15 min what a psychologist achieves in 45 min. That’s not possible. If they worked under different conditions, then maybe the GPs could address much of this [mental disorders] themselves. But they can’t. They can spend 30 min once, but they can’t do that, like, regularly, 12 times, twice a week.* (CMHC 2).

### Having experienced mental health specialists in the team

#### Mental health specialists on the front line can shorten and improve pathways through care

The lowered threshold meant that mental health specialists could intervene while symptoms were relatively manageable. This, several GPs commented, helped patients just below the eligibility threshold to specialist care, for whom they often experienced they did not provide the best service. Participants from all stakeholder groups emphasised that getting in early could prevent conditions from deteriorating:

*In that situation I think it worked like, sort of firefighting really, that I was in contact with that psychologist instead of, because then I could vent things. But that could perhaps have built up if I hadn’t had that contact in that situation, and then maybe, you know, it could have, hypothetically, been harder to handle.* (Patient 7).

Getting in early was also thought to have positive knock-on effects on patients’ wider situations, such as shortening the duration of sick leave:

*They get an appointment the following week and have weekly sessions with the psychologist. After 4–5-6 sessions they’re better and ready to get back to work. And then [they can] use the tools and the knowledge they’ve accumulated with the psychologist, to continue.* (GP7).

A significant part of the psychologists’ work involved “sorting”, as they described it, patients in terms of the type of treatment and service they required and were entitled to. Their specialist experience enabled them to, for example, conduct screening assessments to see if a full specialist assessment was required, or to identify what type of service would be most beneficial to them:

*Certain things are easy for me to do that the GP can’t get done. I can offer a brief assessment with a structured interview, just to clarify, should this go further to a referral for trauma treatment, or can we deal with that here, or refer to the borough’s [name of service]. And do a brief assessment of Asperger’s, which really is very complex, but when you can do it kind of ‘light’, plus [potentially] a referral to a neuropsychologist for confirmation, then you could get these cases dealt with at the GP practice. So I feel that, you know, I can use my specialist expertise and remove some really, like, barriers and potentially long and painful cases, at the GP practice.* (CMHC 4).

CMHC specialists thus advised the GPs, on a case-by-case basis, on whether a patient should be treated at the practice or be referred to the CMHC or to other local services. This was described as increasing the likelihood of the patient’s pathway pointing in the right direction from the outset, avoiding detours or spending time waiting for services for which they would not be eligible. Also, at the GP practice, the psychologists were able to start treatment activities at the patients’ first or second appointment, sometimes without any clear diagnostic picture. At the CMHC, in contrast, patients were, after a few months on a waiting list, subjected to a raft of diagnostic and risk assessments before treatment could commence. Several of the CMHC specialists described this more rapid way of working as “liberating” and that it kept treatment episodes short: most patients were seen 5–10 times. Moreover, given that most of the cases were considered less severe than those they were used to at the CMHC, it was manageable for the CMHC specialists to see substantially more patients per day at the GP practice.

These factors resulted in increased throughput that was pointed out as beneficial by several of the GPs and CMHC specialists. The patient group expressed more mixed views. While they were all grateful for getting into treatment quickly, some experienced the pace of these short treatment episodes as a bit “hectic”, that the clinicians kept “an eye on the clock” or that the treatment seemed a bit simplistic:

*But that gets a bit, like, a “quick fix” for me, really. And then sometimes – and I did let them know – it’s too simple just to come up with “just think differently”. You know, the world isn’t always quite like that.* (Patient 7).

Almost all would have liked to have seen the specialist for longer:

*I wish I had more sessions with [the psychologist]. I don’t think it’s enough with six sessions. Because it took two sessions just to get to know each other.* (Patient 2).

Some patients suggested that follow-up appointments after a few months should routinely be offered to monitor progress.

#### The involvement of specialists can improve referral practices

Many patients were of the opinion that psychological help normally was exceedingly difficult to obtain and that GPs should learn to write more detailed referrals that could help them access it. The clinical participants also recognised referrals from primary to secondary services as potentially problematic and often frustrating, both at the sending and receiving end. Most believed that the collaborative care model would have impact on referrals in different ways. While treating more patients in primary care could *reduce* the number of referrals, an improved identification of need could lead to *more* referrals. However, most participants were concerned with how collaborative care could *improve* processes to ensure timely and comprehensive referrals to the appropriate service for which the patient is eligible. Several contributing factors were described. First, the CMHC specialists often wrote detailed notes that the GPs could consult when writing referrals

*I [usually] wrote a pretty comprehensive note that the GPs could use as part of the referral. So I didn’t write the referral to the CHMC, but they enclosed my notes with [my] assessments and background and, like, why we think, or thought, they ought to be referred to specialist services.* (*CMHC 5*).

Second, these notes could demonstrate that the GP’s view was shared by a specialist, and a few GPs described that this added weight to their referral. Third, the specialists often advised GPs not to refer patients if the underlying problem was one that specialist services could not solve (such as housing or family relationships). In such cases, a referral could create unrealistic expectations and lead to disappointment and depression. Support for the decision that a referral was futile was sometimes all that was required:

*I noticed pretty soon that they [GPs] just needed a kind of confirmation that in this case we’ve done everything. You know, all we* *can* *do, and we don’t expect things to improve. But, you know, they referred simply out of desperation.* (CMHC 6).

Some of the GPs said these ways of working together made them better able to write more focused referrals that included more of the information needed at the receiving end, and that they now were also better able to await situations or explore issues further:

*Now we’re better at, or I am better at, having conversations with [patients] and making my own evaluations before passing them on. That’s after we’ve learnt, you know, understood more of how things are done with regards to referrals, what can be expected from the CMHC. So you think that, OK, here is a patient who doesn’t necessarily need a specialist assessment or something from the CMHC to get any help. This I can handle, so I do it myself rather than referring like [I did] before. (*GP7).

#### Learning through access to complementary skills

Many of the GPs expressed the view that working alongside specialists had increased their ability to detect mental health problems. Several of them said that they had become more targeted in terms of assessing severity and treatment options and that they felt more confident in their own judgement, including the use of screening tools and selection of therapeutic approach but also medication use, dosage and associated blood tests.

For their part, the CMHC specialists had gained a better understanding of how co-morbidities impact mental health. Also, they now had more realistic ideas of the pressures on GPs and their capacity to follow up patients after discharge from specialist services:

*Especially those Monday mornings that are simply chaotic and patients are pouring in, like, completely crazy *[*…*] *I have maybe got a more realistic view of what a GP can help patients with once they've finished at the CMHC. For example, you don’t need to write in the discharge note that “the patient should receive regular supportive therapy from the GP”, because in reality maybe it will be more of a health check than a proper therapeutic session.* (CMHC 2).

The new relationships that were forged were described as an important outcome of working as a collaborative care team. They made it easier to pick up the phone to talk to someone to discuss specific cases or seek advice more generally

*Things go a bit more smoothly when you know each other: you can be bothered to pick up the phone, you know. You prioritise making that phone call a bit sooner when you know who the person is. And [you know] what to expect.* (CMHC 6).

### Barriers to the collaborative care model: the organisation of care

When discussing issues that had either prevented the model from reaching its potential or that could impede its sustainability, most of the issues revolved around the *organisation of care***.** Sub-themes included that logistical issues must be thought through; different modes of working can impede practices and learning, and; that the funding of health care works contrary to collaboration.

### Logistical issues must be thought through

There was a range of practical issues that made project-based collaboration problematic. For GPs, hosting additional members of staff presented logistical issues to make sure there was office space available on the days the CMHC specialists were present. Patients commented that the rooms used were not always ideal as they could be cramped or not provide sufficient privacy. There were also cost implications in terms of rent, IT and other technical equipment, and increased workloads for support staff. For the CMHC, lending out senior clinicians put pressure on other members of staff and on budgets. The clinical participants were clear that for the collaborative care model to be sustainable, permanent solutions to logistical issues must be identified.

### Different modes of working can impede practices and learning

Almost all the clinical participants recognised that, at the outset, they had insufficient knowledge of the practicalities of each other’s everyday working life, and this had led to unrealistic expectations. For example, some GPs initially expected the psychologist to see 12 patients each day. This was soon adjusted to a more realistic level, and the load was settled on 5–6 consultations daily.

During the intervention period, there were situations where the different ways of working could compromise professional practices. One example was how GPs in effect could become gatekeepers for the follow-up of psychotherapeutic treatment:

*And [a GP may say] “Well, the patient just said he didn’t need that appointment”. And that makes us therapists think, well is that avoidance or, like, what is happening to that patient now? And then it’s like, “Oh, that’s a shame because I really wanted a final session.” So, professionally we think very differently.* (CMHC 2).

There were also other difficulties related to the  psychologists not being entirely in charge of their own diary. For instance, if they believed a patient would benefit from 5 to 6 consultations in rapid succession over a couple of weeks, their diaries might already be filled up, and individual patients’ pathways could be prolonged.

As shown above, much of the practical case collaborations happened “in the corridor” due to the time pressures GPs work under. However efficient in daily practice, this ad hoc dimension to the collaboration could limit potential learning on both sides:

*Maybe sometimes, rather than the patient being “mine,” Maybe, together with the GPs, I could have shown a bit of how we talk and work with, for example something as simple as exposure [therapy] for a panic disorder, or, you know how a patient can become less scared of their symptoms, and then maybe the GP could have a go on their own the next time.* (CMHC 2).

### The funding of health care works contrary to collaboration

The ways in which health services are funded was emphasised  as the most fundamental barrier to collaborative care. Many GPs mentioned how the regulations surrounding fee-for-service reimbursements and patient co-payments, both central to their income, created problems: GPs could not charge for treatment he or she did not take part in, and CMHC professionals could not bill on behalf of the GP practice. In some cases, the normal GP fee or fees for collaborative work applied, but in a substantial number of cases no fee was reimbursed and patients were not charged. Similarly, the CMHC specialists could not be reimbursed for treatment not provided within their own service, and the CMHC therefore used significant resources with no immediate return. It was described as ironic that services in effect would be disadvantaged by improving services to their patients. Some of the GPs found it problematic that the GP system – designed to “run like a business” – in effect prevented collaborative work, leading some to express doubt in the wisdom of organising services this way:

*I’m thinking that lots could be done with the GP system [laughs] because I think sitting here and everyone running their own business isn’t really very expedient. And I’d rather be on a regular salary than the way it is now. And maybe have more time, with fewer patients on my list, and more time to work together with other services.* (GP1).

## Discussion

How complex interventions work (or fail to work) depends on myriad issues, including factors at the micro-level [28]. Our qualitative investigation of such factors in the testing out of a collaborative care model for mental health in Oslo, Norway, shows that patients, GPs and CMHC specialists were overall very positive and identified a range of benefits to both patients and services. The main barriers identified were organisational and structural.

### Enabling components to improved accessibility are both operational and relational

According to our participants, the model improved accessibility in different ways, and this was due to two main “active ingredients” [27]. First, *co-location* – as also found in other studies [26] – dramatically increased GP and CMHC specialists’ access to face-to-face contact with each other and to each other’s expertise, enabling ongoing case collaboration. This combination of expertise was accomplished largely through informal meetings but also through patient records or more focused joint work that could encompass both psychiatric and somatic issues and interactions between them. Co-location also lowered the threshold to mental health care, improving patients’ access to specialist input at an early stage.

Second, because the CHMC specialists were highly *experienced mental health specialists,* at consultant level, they could start treatment early or assess and advise on the appropriate type and level of care. This increased throughput and the chances of patients’ pathways going in the right direction from the start, without unnecessary delay. Having mental health specialists at the forefront is not common in the many models for collaborative care described in the literature. Often, these involve nurses [38, 39], counsellors [31, 40] or health assistants [17] as case managers who rely on specialists to assess, treat, prescribe or calibrate medication. Our findings suggest that having mental health specialists on the front line can potentially eliminate some referrals to secondary care but also identify more need. Our participants emphasised that more importantly, it can improve the specificity of referrals, not least because GPs gain knowledge and confidence to deal with complex cases. This corresponds with findings from other studies [16, 17] and addresses the frequently reported problem of GPs having limited access to mental health specialists [6, 10–14]. At an *operational* level then, these two components – co-location and experienced mental health specialists as part of the team – aligned complimentary skill sets, enabled earlier intervention and increased throughput.

In the literature, patients exposed to collaborative care report that they value the opportunities for improved access and patient-centred care [29, 41]. This was mirrored by the patients in our study who, despite expressing a wish for psychological support in the longer term, saw the value of closer collaboration between those involved in their mental health care. For the professionals, learning to know each other by working closely together and being exposed to each other’s practices and expertise increased their insights into how different services operate. This produced more realistic expectations as to each other’s roles, what could be achieved and how. In addition, forging new relationships enabled ongoing collaboration simply because it is easier to contact someone you know. This demonstrates how the *relational* dimension of service delivery may be essential for the implementation of collaborative care [28].

### Barriers to continued collaborative care are largely structural

Our participants also identified barriers to collaborative care. These included logistical and cost implications of having more staff working at the practice and that limited space could produce cramped working conditions and impede patient confidentiality. At the outset there were divergent expectations as to how the model would pan out, such as the number of daily patient consultations a psychologist should have. During the project period, workable solutions were found to these barriers, many of which have been identified in previous studies of collaborative care, including poor communication, sharing of IT or other information systems, location and space issues [25, 26]. Nevertheless, some of the solutions, such as psychologists hopping from office to office, or support staff being stretched, may not be sustainable in the long term. Our findings suggest that, from an operational point of view, collaborative care cannot be an add-on but must be mainstreamed into the planning and resourcing of services.

The most significant barrier identified to the sustainability of collaborative care was the way in which health care is funded. The regulations of fee-for-service and co-payment from patients, two central funding streams underpinning the Norwegian GP system, often prevented GPs from charging either during the intervention period. While the GPs remained committed to the testing of the model, this is obviously not a sustainable solution. Similarly, CMHCs are unlikely to be able, or willing, to carry the cost of lending out senior specialists in the long term. So, while current Norwegian healthcare policy has the explicit aim of increasing collaboration between primary and secondary care and delivering services at the lowest effective care level [3], the split in the organisation and funding of these service levels represents an obstacle to achieving these aims. As was also found in demonstration projects in the US, it may be unlikely that the model is implemented further until novel payment methods make it economically feasible [42].

Our findings reflect the tensions between the different worlds in which healthcare systems simultaneously operate – the clinical, the operational and the financial – and the difficulty in satisfying the demands of all three simultaneously [22]. While stakeholders on the ground can overcome a range of operational (and relational) barriers to provide enhanced clinical pathways, they cannot overcome the structural and financial barriers that stem from the structural organisation of care.

### Strengths and limitations

Our sample included almost all the clinicians involved in the intervention and we applied a broad sampling strategy for the patient sample. This helped us capture a wide range of views and experiences within and across the three groups. As patients were recruited through clinicians, this could have impacted the constitution of the sample. Some patients declined participation. Also, it is possible that, had we been successful in recruiting more participants, particularly patients with mostly negative experiences (of whom psychologists could identify only a handful), we could have gained additional insights. The intervention teams were situated in one area of Oslo, Norway. It is possible that local circumstances mean findings have limited applicability to other settings. However, many of the processes and experiences reported here reflect findings from other settings [25, 26, 29].

## Conclusion

From qualitative interviews with GPs, CMHC specialists and managers, and patients who took part in the adaptation of a collaborative care model in Norway, we found that co-location and having mental health specialists as a core part of the team have the potential to improve accessibility by facilitating professionals’ access to each other. This increased patients’ access to mental health care. Both these dimensions of accessibility directly support current policy that emphasises improving patient access to enhanced patient pathways through collaboration that enable more activities to take place in the primary setting [3]. What was identified as a key barrier, however, was a funding system that in effect penalises such collaborative work. It is difficult to see how successful, sustainable collaboration can be achieved without the government first addressing how to fund collaborative care for patients’ benefit.

## Supplementary information

**Additional file 1.** Interview guide

## Data Availability

Not applicable.

## Abbreviations

ADHD: Attention deficit hyperactivity disorder
CMHC: Community Mental Health Centre
GP: General Practitioner
HFHT-model: Hamilton Family Health Team model
IT: Information technology
PSTD: Post-traumatic stress disorder
RCT: Randomised controlled trial
