# Supplementary material for: Collaborative care for mental health: a qualitative study of the experiences of patients and health professionals
Source: BMC Health Serv Res. 2020 Sep 9;20:844. doi: 10.1186/s12913-020-05691-8 (PMC7487713; doi:10.1186/s12913-020-05691-8)
Supplement: Supplementary file 1 — Additional file 1. Interview guide [file 12913_2020_5691_MOESM1_ESM.docx]

# *Additional file 1 BHSR-D-20-00022*

**English translation of topic guide for individual interviews with GPs and CMHC specialists.**

Prompt for examples throughout.

At the personal level:

- General experiences of working according to the model; how it was experienced and what it entailed for the individual GP/CHMC specialist
- Changes to individual work practice; experiences and views of such change
- Individual learning and skills development and potential impact on interaction with and treatment of patients
- Views on individual practice vis-à-vis patients in the target population during the project period in terms of
  - Identification and diagnostics
  - Treatment
  - Referral practices within and between services
  - Collaboration between services at municipal and specialist levels (prompt for relevant local services)

At the level of the GP practice/CHMC

- Perceptions/views on whether and how working according to the model improved the service offered by the practice
- Aspects of the model that worked well and which ones worked best; perceived reasons why
- Aspects of the model that were challenging and problematic; perceived reasons why
- Whether model implementation impacted on referrals between services
- What, if anything could have improved the model
- How the structural organisation of care impacted on model implementation
- Whether the GP practice/CMHC will continue with elements of the model; why/why not

**English translation of topic guide for individual interviews with patients**

Prompt for examples throughout.

- How long the patient has been with the current GP
- How long the patient has been treated for the current mental health problem
- View on how GPs best can help people with the patient’s current mental health problem. Prompt for
  - Access
  - Collaboration with other services
- Experiences and evaluation of the service received at the GP practice during the model implementation period. Prompt for
  - What was offered
  - Advantages
  - Disadvantages
- Views and experiences of how the different services at municipal and specialist levels have collaborated about the patient’s care during the last year (prompt for relevant local services)
  - What worked well
  - What did not work well
  - Areas of improvement
- Views of whether the services received, including the collaboration between them, were different that it was prior to the project
  - Better, worse, the same
- Whether the patient gets the help s/he feels is needed
- Views of what could be improved in their care
